# Cerebral blood flow regulation, central arterial stiffness and traumatic brain injury: Effects of aerobic exercise training

**DOI:** 10.1113/EP093330

**Published:** 2026-02-05

**Authors:** Tsubasa Tomoto, Kan Ding, C. Munro Cullum, Rong Zhang

**Affiliations:** ^1^ Human Informatics and Interaction Research Institute National Institute of Advanced Industrial Science and Technology (AIST) Tsukuba Ibaraki Japan; ^2^ Integrated Research Center for Self‐Care Technology National Institute of Advanced Industrial Science and Technology (AIST) Tsukuba Ibaraki Japan; ^3^ Institute for Exercise and Environmental Medicine Texas Health Presbyterian Hospital Dallas Dallas Texas USA; ^4^ Departments of Neurology, Internal Medicine University of Texas Southwestern Medical Center Dallas Texas USA; ^5^ Departments of Psychiatry, Internal Medicine University of Texas Southwestern Medical Center Dallas Texas USA; ^6^ Departments of Neurological Surgery, Internal Medicine University of Texas Southwestern Medical Center Dallas Texas USA; ^7^ Biomedical Engineering, and Internal Medicine University of Texas Southwestern Medical Center Dallas Texas USA; ^8^ Internal Medicine University of Texas Southwestern Medical Center Dallas Texas USA

**Keywords:** aerobic exercise training, age, arterial stiffness, cerebral blood flow, traumatic brain injury

## Abstract

Advanced age is the strongest risk factor for Alzheimer's disease and related dementias (ADRDs). Traumatic brain injury (TBI) has also been recognized as a risk factor for ADRD, potentially contributing to an earlier onset of the disease. Thus, elucidating the mechanisms underlying brain ageing and TBI is critical for developing strategies to preserve brain health. Accumulating evidence indicates that arterial ageing, manifested as increased central arterial stiffness, is closely associated with cerebral blood flow (CBF) dysregulation and brain ageing, whereas improvement of CBF regulation through aerobic exercise training contributes to brain health. Emerging studies suggest that central arterial stiffness is elevated after TBI but can be ameliorated by aerobic exercise training, which benefits TBI recovery. In this brief review, we summarize evidence demonstrating that (1) age‐ or TBI‐related increases in central arterial stiffness are associated with CBF dysregulation, and (2) aerobic exercise training improves CBF regulation by reducing central arterial stiffness in both healthy older adults and individuals with chronic TBI. Collectively, these findings support the hypothesis that central arterial stiffness impacts CBF regulation and highlight aerobic exercise as a promising intervention for preserving brain health in ageing and after TBI.

## Abstract (highlights)

- **What is the topic of this review?**
  This review summarizes evidence demonstrating that ageing‐ and traumatic brain injury (TBI)‐related central arterial stiffening is associated with impaired cerebral blood flow (CBF) regulation, and that aerobic exercise training improves CBF regulation by modifying central arterial stiffness in both healthy individuals and those with chronic TBI.
- **What advances does it highlight?**
  Preliminary findings suggest mechanisms linking central arterial stiffness to CBF regulation and that aerobic exercise is a promising intervention to preserve brain health in older adults and individuals with chronic TBI.

## 1 INTRODUCTION

The incidence of dementia continues to increase with the global ageing population, and advanced age is the primary risk factor for Alzheimer's disease and related dementias (ADRDs). Although recently developed anti‐amyloid therapies for Alzheimer's disease (AD) modestly reduced cognitive decline and the related pathological progression, curative treatments remain unavailable (Alzheimer's Association, 2024). Thus, a deeper understanding of brain ageing, related risk factors and their relationship to dementia prevention is essential for developing strategies to preserve brain health and delay the onset or progression of ADRD (Livingston et al., 2024).

The transformation from healthy brain ageing to AD is multifactorial, involving a combination of genetic, lifestyle and environmental factors accumulated over a life course (Alzheimer's Association, 2024). Recently, traumatic brain injury (TBI) has been identified as an important risk factor for ADRD, potentially contributing to an earlier onset of cognitive decline (Livingston et al., 2024; LoBue et al., 2019). Beyond its acute effects, TBI is increasingly recognized as a chronic condition with long‐term consequences, including an elevated risk of cardiovascular disease (Izzy et al., 2023) and other neurological disorders (Maas et al., 2022). Notably, a history of TBI with reported loss of consciousness has been associated with an earlier onset of AD by 2–3 years compared with individuals without a history of TBI (Schaffert et al., 2018). Although the underlying mechanisms for this association are not known, primary injuries from TBI with severities ranging from mild to severe can involve traumatic axonal damage and cerebrovascular injury, which may contribute to secondary brain injury characterized by white matter damage, cerebral hypoperfusion, autonomic dysfunction and blood pressure dysregulation (Maas et al., 2022). The cerebral blood flow (CBF) dysregulation and cardiovascular autonomic dysfunction after TBI may therefore initiate and contribute to long‐term neurodegeneration that overlaps with and/or accelerates age‐related brain pathology (Kisler et al., 2017; Ramos‐Cejudo et al., 2018).

Adequate CBF and its regulation are essential for maintaining normal brain function. Cerebral hypoperfusion has emerged as an important risk factor for age‐related cognitive decline (Kisler et al., 2017) and is one of the earliest pathophysiological changes preceding AD pathology (Iturria‐Medina et al., 2016). In addition, CBF dysregulation following moderate to severe TBI and repeated mild TBI has been associated with elevated risks of cognitive impairment and emotional difficulties (Maas et al., 2022) as well as brain β‐amyloid and tau deposition (Ramos‐Cejudo et al., 2018). These observations collectively suggest that CBF dysregulation may represent a mechanistic link between TBI and ADRD (Ramos‐Cejudo et al., 2018).

Arterial ageing, manifested as central arterial stiffening, is closely linked to CBF dysregulation. Central arterial stiffening increases systemic and cerebral arterial pulsatility, thereby exposing cerebral small blood vessels to augmented mechanical stress (Thorin‐Trescases et al., 2018). This process may lead to endothelial dysfunction, elevated cerebrovascular resistance (CVR), and cerebral hypoperfusion, which likely contribute to age‐related cognitive decline in older adults (Tarumi et al., 2025; Thomas et al., 2021; Tomoto & Zhang, 2024). Importantly, elevations in central arterial stiffness, cerebral arterial pulsatility, CVR and cerebral hypoperfusion are exacerbated in individuals with a chronic stage of moderate to severe TBI compared with non‐TBI adults (Rim et al., 2025; Thomas et al., 2021, 2022).

**FIGURE 1:**
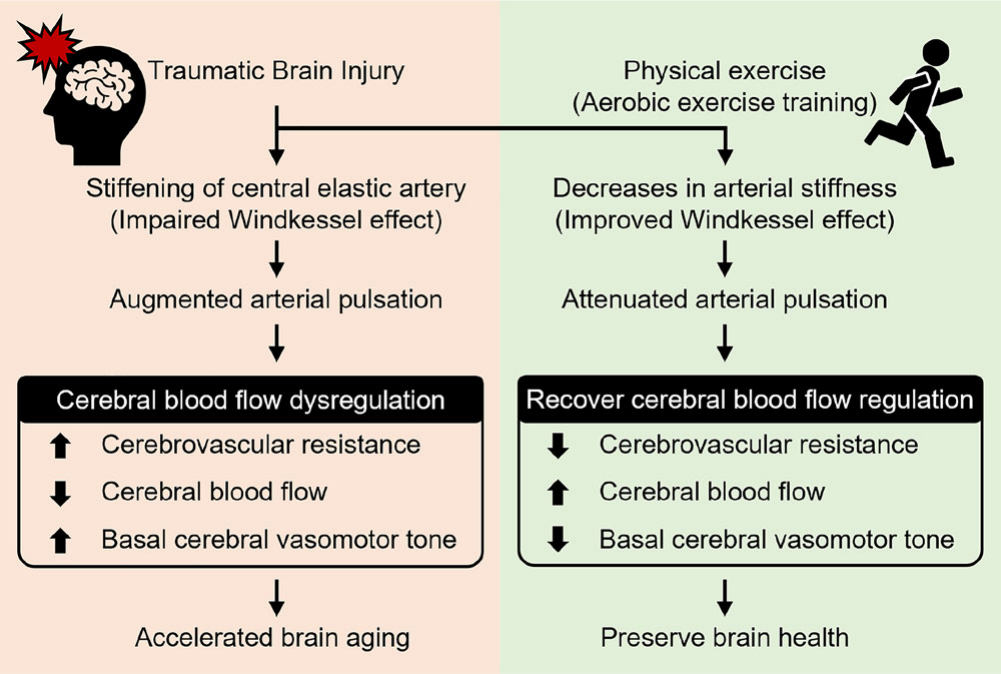
A proposed hypothesis of the Windkessel effect of central elastic arteries on cerebral blood flow regulation after traumatic brain injury and how aerobic exercise training may ameliorate the effects of traumatic brain injury on cerebral blood flow regulation.

Mounting evidence suggests that aerobic exercise training improves CBF regulation and may help prevent or attenuate cognitive decline in middle‐aged and older adults (Tarumi et al., 2025; Tomoto & Zhang, 2024). Although the mechanisms remain incompletely understood, reductions in central arterial stiffness have been observed following aerobic exercise in cognitively normal older adults and patients with mild cognitive impairment (MCI) (Barnes et al., 2021; Tanaka, 2019; Tarumi et al., 2025; Tomoto & Zhang, 2024). We proposed that decreased central arterial stiffness may reduce systemic and cerebral arterial pulsatility and CVR, thereby increasing CBF and preserving cognitive function in older adults (Tarumi et al., 2025; Tomoto & Zhang, 2024). This hypothesis may also be relevant to individuals with TBI in that aerobic exercise training may improve CBF regulation and reduce the risk of ADRD (Figure 1).

Besides reductions in central arterial stiffness, aerobic exercise training improves endothelial function, dynamic cerebral autoregulation, neurovascular coupling, and cerebral vasomotor reactivity to CO_2_, which may improve brain health after TBI, related to or independent of reduction in central arterial stiffness (Tomoto & Zhang, 2024).

In this brief review, we provide evidence that (1) age‐ and TBI‐related central arterial stiffening is associated with CBF regulation, and (2) aerobic exercise training improves CBF regulation by modifying central arterial stiffness in both healthy ageing individuals and those with chronic TBI. Collectively, these findings suggest a mechanistic link between central arterial stiffness and CBF regulation and that aerobic exercise preserves brain health in the context of ageing and TBI.

## 2 ARTERIAL AGEING, CEREBRAL BLOOD FLOW AND TRAUMATIC BRAIN INJURY

Arterial ageing is characterized by stiffening of the central elastic arteries (e.g., aorta and carotid arteries), a key determinant of augmented arterial pulsatility that contributes to CBF dysregulation (Thorin‐Trescases et al., 2018). Importantly, TBI‐induced cardiovascular dysfunction may further elevate central arterial stiffness, thereby influencing CBF regulation (Ding et al., 2020; Thomas et al., 2021, 2022). Below, we discuss the physiological mechanisms, assessment methods, and the impact of central arterial stiffness on CBF regulation.

### 2.1 Central elastic artery stiffness and cerebral arterial pulsatility

The central elastic arteries have the ability to buffer arterial pulsations induced by the stroke volume from the left ventricle and provide steady blood flow to peripheral vascular beds, referred to as the Windkessel effect (Belz, 1995). This buffering capacity is primarily mediated by elastin, collagen and smooth muscle within the arterial wall (Herzog et al., 2025). Elastin, in particular, buffers the majority of the pulsatile mechanical stress generated by intermittent left ventricular ejection. During systole, the arterial wall expands to accommodate stroke volume, thereby reducing the transmission of excessive systolic pressure into the downstream microcirculation (Thorin‐Trescases et al., 2018). In diastole, the arterial wall recoils as stored elastic energy is released, maintaining diastolic pressure and continuous peripheral perfusion (Belz, 1995). Through this arterial wall movement, the Windkessel effect of central elastic arteries protects the brain from potentially damaging excessive arterial pulsatility while maintaining efficient tissue perfusion.

Several methodologies have been utilized to evaluate the elastic properties of central arteries in humans. Carotid–femoral pulse wave velocity (cfPWV), considered the gold standard for assessing central arterial stiffness, is calculated based on the distance between the carotid and femoral arteries and the transit time of an arterial pulse wave between these sites (Herzog et al., 2025). Thus, cfPWV reflects the integrated stiffness of multiple aortic segments. In contrast, carotid arterial stiffness (e.g., the carotid β‐stiffness index and arterial compliance) is derived from ultrasound‐based measurements of carotid lumen diameter changes with arterial pulse pressure obtained via applanation tonometry at the common carotid artery (Herzog et al., 2025). Thus, carotid arterial stiffness is likely to be more relevant to assess the impacts of central arterial stiffness on CBF regulation (Tomoto et al., 2021b, 2023b).

Age‐related stiffening of central arteries is attributed to elastin fragmentation, increased collagen deposition and alterations in vascular smooth muscle tone (Herzog et al., 2025). The central arterial stiffening impairs the Windkessel effect, leading to elevated pulsatile arterial pressure and blood flow that can damage small cerebral blood vessels. The brain is particularly vulnerable to excessive arterial pulsatility due to its low vascular resistance and high perfusion, allowing increased pulsatile energy to penetrate into the microcirculation and contribute to cerebral small blood vessel disease (CVSD) (Thorin‐Trescases et al., 2018). CSVD, often manifested as white matter hyperintensities (WMHs) on MRI, is linked to age‐related brain atrophy as well as cognitive decline (Thorin‐Trescases et al., 2018). Notably, we observed that cerebral arterial pulsatility mediated the associations between increased carotid arterial stiffness and WMH after controlling for age, sex and mean arterial pressure (Tomoto et al., 2023b). Collectively, these findings suggest that the Windkessel effect of the central artery on buffering systemic arterial pulsations is associated with cerebral arterial pulsatility, which is linked to brain white matter structural changes in brain ageing.

### 2.2 Cerebral blood flow and cerebrovascular resistance

A sufficient and continuous blood supply of oxygen and energy substrate (i.e., glucose) to the brain is necessary to maintain normal neuronal activity. Although the brain accounts for only 2–3% of total body mass in humans, it receives approximately 15% of cardiac output and consumes about 20% of available oxygen under resting conditions (Claassen et al., 2021). The brain's high metabolic demand, coupled with its limited energy reserves, underscores the critical role of CBF in ensuring sufficient oxygen and nutrient supply (Kisler et al., 2017). To accommodate this demand, CVR is lower relative to other organs. Notably, a substantial portion of CVR is regulated by the large extracranial arteries and pial arterioles (Claassen et al., 2021). Therefore, proper adjustment of CVR in response to blood flow demand is essential for maintaining normal brain function.

Several non‐invasive imaging techniques have been used to assess volumetric CBF, CBF velocity (CBFV), and brain perfusion (Fantini et al., 2016). Phase‐contrast magnetic resonance imaging (PC‐MRI) and color‐coded duplex ultrasonography (CDUS) are commonly used to quantify both volumetric CBF and CBFV in the extracranial arteries supplying the brain, such as the internal carotid (ICA) and vertebral (VA) arteries. In addition, transcranial Doppler ultrasonography (TCD) has been used to measure CBFV in the intracranial vessels, such as the middle cerebral artery (MCA), providing an indirect index of changes in CBF assuming the diameters of the insonated arteries remain constant (Fantini et al., 2016). Arterial spin labeling (ASL) MRI has been utilized to quantify both global and regional brain perfusion. In this regard, our recent findings showed that total CBF, as well as CBF normalized to brain volume, exhibited significant correlations across CDUS, PC‐MRI and ASL. Similarly, blood flow velocity measurements in the ICA, VA and MCA were correlated among CDUS, PC‐MRI and TCD, despite substantial inter‐individual variability (Tomoto et al., 2023a).

Previous studies have reported age‐related declines in CBF, which may arise from reduced cerebral metabolism, CBF dysregulation or a combination of both (Claassen et al., 2021). One proposed mechanism implicates CBF dysregulation, whereby age‐related central arterial stiffening and increased arterial pulsatility subject cerebral arterioles and capillaries to greater mechanical stress. This, in turn, may contribute to endothelial dysfunction, vasoconstriction, elevated CVR and reduced CBF in older adults (Thorin‐Trescases et al., 2018; Tomoto & Zhang, 2024). Notably, a longitudinal study in older individuals with and without a clinical diagnosis of AD demonstrated that elevations in CVR preceded reductions in CBF and predicted the onset of AD independently of metabolic alterations (Yew et al., 2017). These findings align with the evidence indicating that CBF dysregulation, manifested as cerebral hypoperfusion, occurs prior to the development of AD pathology in ageing populations (Iturria‐Medina et al., 2016).

Cerebral hypoperfusion, elevated CVR and central arterial stiffening are increasingly recognized as vascular contributors to age‐related cognitive decline and dementia (Herzog et al., 2025). We recently tested this hypothesis by showing that carotid arterial stiffness was associated with reduced CBF and increased CVR in patients with MCI, a prodromal stage of dementia (Tomoto et al., 2020). Compared with age‐matched cognitively normal older adults, patients with MCI exhibited lower CBF and higher CVR. Total CBF assessed by CDUS was negatively associated with carotid arterial stiffness, and CVR was positively associated with carotid systolic pressure after adjustment for age, sex, body mass index and MCI status. Cerebral pulsatility at the MCA was positively associated with carotid pulse pressure. Importantly, lower diastolic CBFV at the MCA and the ICA was associated with higher carotid arterial stiffness and lower total CBF, implicating an impaired Windkessel effect of the central artery during diastole in cerebral hypoperfusion (Ashley et al., 2025; Tomoto et al., 2020). Alternatively, the presence of AD pathology, such as brain β‐amyloid and tau in patients with MCI, may contribute to cerebral vasoconstriction, leading to CBF dysregulation (Pasha et al., 2020).

### 2.3 Cerebral blood flow regulation and central arterial stiffness in traumatic brain injury

TBI is clinically heterogeneous, and the nature and magnitude of CBF dysregulation may vary by injury severity (mild, moderate and severe) and time since injury (acute or subacute versus chronic) (Maas et al., 2022). Acute moderate‐to‐severe TBI is often characterized by marked disturbances in brain perfusion and CBF regulation, whereas mild TBI may present with subtle CBF dysregulation, which may persist in individuals associated with repeated mild TBI (Patricios et al., 2023). We will specify TBI severity and temporal stages where possible when summarizing the evidence below.

Despite increased research on TBI, the pathophysiological mechanisms of brain injury, cerebrovascular injury and their potential impacts on long‐term neurocognitive function remain elusive (Maas et al., 2022; Patricios et al., 2023; Sandsmark et al., 2019). TBI occurs when an impulsive force is transmitted to the brain, resulting in stretching and disruptions of neuronal membranes, which may initiate a hyper‐neurometabolic cascade involving depolarization of neurons and indiscriminate glutamate release with excessive intracellular ionic flux (Giza & Hovda, 2014). This disruption of cellular homeostasis causes the ion pumps to use more ATP, with a significant increase in glucose metabolism (Giza & Hovda, 2014). This injury‐related hypermetabolism likely occurs in the setting of reduced CBF, resulting in a disparity between glucose supply and demand, which may trigger a cellular energy crisis leading to cellular damage and cell death (Giza & Hovda, 2014).

Acute traumatic cerebrovascular injury is a frequent, if not universal, feature after moderate to severe TBI, which may also occur after mild TBI, particularly in repeated mild TBI. Primary cerebrovascular injury comprises vasospasm of larger cerebral arteries, trauma‐induced vascular injury at the arteriole and capillary levels, and damage to the blood–brain barrier and endothelium, which may be related to shear stress forces during TBI (Sandsmark et al., 2019). Secondary injury processes include altered brain metabolism and metabolic waste clearance, and neuroinflammation, which further leads to cerebral hypoperfusion, impaired neurovascular coupling and impaired cerebral autoregulation (Giza & Hovda, 2014; Sviri et al., 2009). Evidence from TBI animal models has demonstrated that cerebrovascular injury leads to amyloid‐β and tau production/aggregation, abnormal inflammatory response and a reduction of brain clearance during the acute stage (Ramos‐Cejudo et al., 2018). Clinical evidence indicates that acute CBF dysregulation measured by brain perfusion and cerebral autoregulation after severe TBI was associated with poor functional outcome at 6 months (Preiksaitis et al., 2016; Sviri et al., 2009). As noted above, beyond its acute effects, moderate to severe TBI and repeated mild TBI are recognized as a chronic condition with potential long‐term consequences, including an elevated risk of AD and cardiovascular disease in some individuals (Ellingson et al., 2024; Izzy et al., 2023).

Emerging evidence suggests that, in addition to CBF dysregulation, central arterial stiffness may increase following TBI (Rim et al., 2025; Tomoto et al., 2022). In a longitudinal case–control study, collegiate American football athletes with sport‐related concussion showed persistently elevated blood pressure and central arterial stiffness assessed by cfPWV over 2 years compared with a control group (Rim et al., 2025). In a cross‐sectional study of patients with chronic moderate‐to‐severe TBI (>6 months), carotid arterial stiffness tended to be increased, and CVR and systolic blood pressure were significantly elevated relative to age‐matched healthy adults (Tomoto et al., 2022). Of note, higher carotid arterial stiffness was associated with greater pulsatile CBF and CVR (Tomoto et al., 2022). In the same cohort, lower regional CBF in the hippocampus and rostral anterior cingulate, measured by ASL MRI, was associated with affective symptoms after TBI, including fatigue, anxiety, depression and sleep impairment (Thomas et al., 2021). Furthermore, impaired CBF regulation assessed by transfer functional analysis was associated with persistent cognitive impairment following TBI (Ding et al., 2020). Collectively, these findings supported the proposed hypothesis in Figure 1 and highlighted the need for further longitudinal studies to examine the relationship between central arterial stiffness, CBF dysfunction and brain health after TBI.

Elevated carotid arterial stiffness after TBI is likely to be multifactorial, though the underlying mechanisms remain largely unknown. In our previous study (Tomoto et al., 2022), cohorts were matched for age, body mass index and fitness level, accounting for these factors as potential explanations for the observed higher carotid arterial stiffness and CBF dysregulation among individuals with chronic moderate to severe TBI. This observation suggests that alternative mechanisms intrinsic to TBI may underlie the elevation in central arterial stiffness. First, the neck flexion–hyperextension that occurs at the time of injury may damage the carotid artery (Martin et al., 1991). Animal studies indicate that overstretching of the arterial wall disrupts collagen and elastin cross‐linkages, thereby altering vascular stiffness (Lillie & Gosline, 2007). Second, autonomic dysfunction and reduced baroreflex sensitivity after moderate‐to‐severe TBI may be related to increases in central arterial stiffness (Ellingson et al., 2024). The baroreflex regulates short‐term blood pressure fluctuations through mechanical activation of arterial baroreceptors located in the carotid sinus and aortic arch, providing feedback control of heart rate and systemic vascular resistance. In addition, TBI‐induced sympathetic overactivity may lead to increased vascular tone and elevated blood pressure, thereby elevated central arterial stiffness, which in turn may reduce arterial distensibility and diminish baroreflex sensitivity (Khalid et al., 2019). Notably, baroreflex sensitivity has also been linked to CBF regulation in healthy adults (Tomoto et al., 2021b). Further investigations are warranted to understand the mechanisms that may account for increased central arterial stiffness following TBI.

## 3 EFFECTS OF AEROBIC EXERCISE TRAINING ON ARTERIAL STIFFNESS AND CEREBRAL BLOOD FLOW REGULATION

Interventions aimed at reducing central arterial stiffness hold potential for improving CBF regulation and lowering the risk of ADRD in both healthy adults (Herzog et al., 2025) and individuals with TBI (Maas et al., 2022). Although previous studies have reported decreases in central arterial stiffness by aerobic exercise training (Tanaka, 2019), effects of aerobic exercise training on CBF regulation are inconclusive (Bliss et al., 2021). Below, we discuss the potential relationship between aerobic exercise training‐induced reduction of central arterial stiffness and consequent improvement of CBF regulation in older adults (Tomoto et al., 2021a, 2023c) and individuals with a chronic stage of moderate to severe TBI (Tomoto et al., 2022).

### 3.1 Central arterial stiffness and cerebral blood flow

Aerobic exercise constitutes a well‐established lifestyle intervention that improves cardiovascular function and may benefit brain health in healthy adults and individuals recovering from TBI. During aerobic exercise, rhythmic contractions of large skeletal muscles elicit profound systemic physiological responses, including elevations in cardiac output and oxygen consumption to satisfy increased metabolic demands. The brain coordinates motor activity and modulates cardiorespiratory function to meet the increased metabolic demand. With sustained aerobic exercise training, the cumulative impacts of physiological stimuli may improve blood pressure control and CBF regulation (Ogoh & Ainslie, 2009; Tan et al., 2014). Notably, moderate‐intensity aerobic exercise has been demonstrated to improve arterial health by attenuating central arterial stiffness and improving CBF regulation (Tomoto & Zhang, 2024). Over time, these vascular adaptations may initiate a cascade of physiological processes to preserve brain health (Bliss et al., 2021; Herzog et al., 2025).

Regular aerobic exercise decreases central arterial stiffening and contributes to improved CBF regulation in healthy adults (Barnes et al., 2021; Tarumi et al., 2025; Tomoto & Zhang, 2024). The reduced central arterial stiffness with moderate‐intensity aerobic exercise training has been attributed partially to a decrease in the vascular smooth muscle tone, which is regulated by the vessel wall endothelial function related to nitric oxide bioavailability (Tanaka, 2019). Thus, exercise‐induced reduction in central arterial stiffness, arterial pulsation and improvement in cerebral endothelial functions may collectively contribute to decreases in CVR and increases in CBF (Tomoto & Zhang, 2024).

Despite the evidence supporting the effects of aerobic exercise training on CBF regulation, the overall impacts on brain health are inconclusive (Bliss et al., 2021; Smith et al., 2021). A recent systematic review and meta‐analysis of the effects of cardiorespiratory fitness and aerobic exercise training on CBF reported that higher cardiorespiratory fitness was associated with higher CBFV measured at the MCA using TCD among normal healthy adults in cross‐sectional studies (Smith et al., 2021). However, intervention involving moderate‐intensity aerobic exercise for a duration of 2–12 months had little influence on the MCA CBFV and global brain perfusion measured using MRI ASL (Smith et al., 2021). It should be noted that no change in CBFV does not preclude increases in volumetric CBF if resting cerebral conduit artery diameter increases with exercise training. Consistent with this possibility, Lapidaire et al. (2023) reported increased resting ICA and MCA (M1) lumen diameters following a 16‐week supervised aerobic exercise training, suggesting that velocity‐based measures may underestimate training‐induced improvements in CBF. In this context, to gain insights into the effect of aerobic exercise training on CBF, CVR and central arterial stiffness, we conducted a 1‐year, open‐label, parallel randomized controlled trial in both cognitively normal older adults and patients with MCI. The effects of moderate‐to‐vigorous aerobic exercise training on CBF, CVR and arterial stiffness were compared with an activity control group of stretching and toning interventions (Tomoto et al., 2021a, 2023c).

We found that the 1‐year progressive, moderate‐to‐vigorous aerobic exercise training increased global CBF and decreased CVR and carotid arterial stiffness in both cognitively normal older adults (Tomoto et al., 2023c) and patients with MCI (Tomoto et al., 2021a). Of note, aerobic exercise‐induced increases in CBF were mainly due to the increased ICA blood flow in both cognitively normal older adults and patients with MCI. Furthermore, decreased carotid arterial stiffness was associated with increased CBF in both cognitively normal older adults and patients with MCI, and with decreased CVR in cognitively normal older adults. The mediation analysis showed that the negative associations between changes in cardiorespiratory fitness and CVR were mediated by the reduction of carotid arterial stiffness in cognitively normal older adults. In patients with MCI, CBF pulsatility was reduced in the aerobic exercise group, and a mediation analysis showed that the positive associations between change in cardiorespiratory fitness and CBF were mediated by a reduction in carotid arterial stiffness and CBF pulsatility. Collectively, these observations suggest that aerobic exercise training reduces carotid arterial stiffness, resulting in improved CBF regulations (Tomoto & Zhang, 2024).

### 3.2 Cerebral blood flow regulation in traumatic brain injury

Individuals with chronic moderate‐to‐severe TBI tend to have lower levels of physical activity, and decreased mobility is associated with increased mortality after TBI. Several studies have suggested that low‐to‐moderate intensity aerobic exercise training may counteract physical deconditioning in this population (Maas et al., 2022; Pham et al., 2023). Alternatively, strict rest from all activities (i.e., cocooning) after mild TBI may increase the risk of persisting concussion symptoms (Leddy et al., 2018). In this regard, low‐to moderate‐intensity aerobic exercise training may also improve CBF in patients with chronic TBI (Maas et al., 2022) and individuals in those recovering from mild TBI (Patricios et al., 2023). Because CBF dysregulation may underlie some clinical symptoms across acute, subacute and chronic phases after TBI, restoring CBF regulation has been proposed as a therapeutic target for persisting post‐concussion symptoms (Maas et al., 2022; Tan et al., 2014; Worts et al., 2019). However, given the small sample size in these previous studies and lack of longitudinal studies, the effects of exercise training as well as intensity of exercise on CBF regulation after TBI, including sport‐related concussions, need to be further elucidated (Maas et al., 2022; Tan et al., 2014; Worts et al., 2019).

We recently conducted a pilot study to investigate the effects of moderate‐intensity aerobic exercise training versus an active control condition involving stretching and toning on CBF, CVR and carotid arterial stiffness in patients with chronic (>6 months) moderate‐to‐severe TBI (Tomoto et al., 2022). We observed that increases in cardiorespiratory fitness were correlated with decreases in carotid arterial stiffness. Moreover, we observed a trend toward improved carotid arterial stiffness in the aerobic exercise training group compared with the active control group (Tomoto et al., 2022). CBF increased in both groups after the 3‐month intervention; however, there was no significant intervention effect on CVR. The magnitude of carotid arterial stiffness reduction after 3 months of aerobic exercise training was comparable to that reported in previous studies in healthy adults (Tanaka, 2019). These findings suggest that moderate‐intensity aerobic exercise training exerts similar beneficial effects on carotid arterial stiffness in individuals with chronic TBI and support the hypothesis presented in Figure 1.

In this pilot study, we found that decreased carotid arterial stiffness was associated with attenuated CBF pulsatility following 3 months of moderate‐intensity aerobic exercise training (Tomoto et al., 2022). This finding suggests that exercise‐induced improvement of the Windkessel effect of the carotid artery may buffer cerebral arterial pulsations and thereby reduce cerebral vasoconstrictor tone. Nevertheless, it remains unclear why improvements in carotid arterial stiffness did not translate into reductions in CVR and increases in CBF. One plausible explanation is that sustained reductions in central arterial stiffness may need to be maintained over a longer duration to exert measurable effects on cerebrovascular regulation. Thus, more prolonged exercise interventions (e.g., ≥1 year) may be required to capture these adaptations (Tomoto et al., 2021a, 2023c).

Resistance exercise is also commonly prescribed and may influence central arterial stiffness. A meta‐analysis indicates that high‐intensity resistance training can increase arterial stiffness in young healthy adults, whereas low‐ to moderate‐intensity protocols generally have neutral effects in middle‐age and older healthy adults (Cortez‐Cooper et al., 2008; Zhang et al., 2021). In contrast, combined aerobic and resistance training has been reported to reduce arterial stiffness in older adults (da Silva et al., 2024). In individuals with TBI, data on the effects of resistance exercise training on central arterial stiffness are limited, and current evidence does not support recommendations of resistance training to reduce central arterial stiffness in individuals with TBI (Leddy et al., 2018; Tan et al., 2014; Worts et al., 2019).

## 4 CLINICAL IMPLICATIONS

Dementia represents one of the greatest societal and scientific challenges of the 21st century. Beyond its profound impact on the health‐related quality of life of affected individuals and their families, the economic burden on patients, caregivers and healthcare systems is substantial and continues to rise. Evidence indicates that TBI is a risk factor for ADRD, potentially contributing to the earlier onset and exacerbation of brain pathology. Thus, a deeper understanding of brain ageing and TBI‐related changes as well as their relationship to ADRD is essential for developing strategies to preserve brain health and delay the onset or progression of ADRD (Livingston et al., 2024).

In this review, we have summarized evidence that arterial ageing, manifested by increased central arterial stiffness, is associated with elevated CBF pulsatility, increased CVR and reduced CBF. Importantly, 1 year of progressive, moderate‐to‐vigorous aerobic exercise training increased cardiorespiratory fitness, reduced carotid arterial stiffness, decreased CVR and improved CBF in older adults. In this regard, it should be noted that aerobic exercise training in individuals with chronic moderate to severe TBI or repeated mild TBI should be carefully considered and tailored to meet the needs of the individuals, which often prioritizes low‐to‐moderate intensity with gradual, symptom‐limited progression. In individuals with chronic moderate to severe TBI, 3 months of moderate‐intensity aerobic exercise decreased carotid arterial stiffness, which was associated with reduced cerebral arterial pulsatility. Collectively, these findings support the hypothesis that reducing central arterial stiffness is an important mechanism by which aerobic exercise training improves CBF regulation and contributes to brain health. Further studies are warranted to confirm and extend these observations and to elucidate the vascular mechanisms linking aerobic exercise and brain health in both older adults and individuals with TBI.

## 5 CONCLUSIONS

This review has summarized the evidence supporting the hypothesis that age‐ and TBI‐related increases in arterial stiffness and arterial pulsatility are linked to CBF dysregulation. Importantly, evidence indicates that aerobic exercise training reduces carotid arterial stiffness and is associated with improved CBF regulation in healthy older adults, patients with MCI, as well as in individuals with chronic TBI. Collectively, these findings suggest that improvement in central arterial stiffness is an important, potentially modifiable mechanism by which aerobic exercise training enhances CBF regulation and preserves brain health including in individuals who have sustained TBI.

## AUTHOR CONTRIBUTIONS

Tsubasa Tomoto drafted the manuscript. Rong Zhang, Kan Ding and Munro Cullum revised the manuscript. All authors have read and approved the final version of this manuscript and agree to be accountable for all aspects of the work in ensuring that questions related to the accuracy or integrity of any part of the work are appropriately investigated and resolved. All persons designated as authors qualify for authorship, and all those who qualify for authorship are listed.

## CONFLICT OF INTEREST

None declared.
